# The effects of OPRM1 118A>G on methadone response in pain management in advanced cancer at end of life

**DOI:** 10.1038/s41598-024-54009-9

**Published:** 2024-02-10

**Authors:** Larisa M. Haupt, Alison Haywood, Heidi G. Sutherland, Chieh Yu, Cassie L. Albury, Anushka Pharasi, Mathew Zunk, Rani George, Lyn R. Griffiths, Phillip Good, Janet Hardy

**Affiliations:** 1 Centre for Genomics and Personalised Health, Genomics Research Centre, School of Biomedical Sciences, Queensland University of Technology, Brisbane, Australia; 2 ARC Training Centre for Cell and Tissue Engineering Technologies, Queensland University of Technology (QUT), Brisbane, Australia; 3 Max Planck Queensland Centre for the Materials Sciences of Extracellular Matrices, Brisbane, Australia; 4 School of Pharmacy and Medical Sciences, Griffith University, Gold Coast, Australia; 5 Mater Research Institute-The University of Queensland, Brisbane, Australia; 6 Department of Cell and Tissue Biology, University of California, San Francisco, USA; 7 Cancer Trials Unit, Division of Cancer Services, Metro South Health, Brisbane, Australia; 8 Department of Palliative Care, St Vincent’s Private Hospital, Brisbane, Australia; 9 Department of Palliative and Supportive Care, Mater Health, Brisbane, Australia

**Keywords:** Genetics, Molecular medicine, Oncology

## Abstract

Cancer pain is the most feared symptom at end of life. Methadone has advantages over other opioids but is associated with significant variability in clinical response, making dosing challenging in practice. OPRM1 is the most studied pharmacogene associated with the pharmacodynamics of opioids, however reports on the association of the A118G polymorphism on opioid dose requirements are conflicting, with no reports including methadone as the primary intervention. This association study on *OPRM1* A118G and response to methadone for pain management, includes a review of this genetic factor’s role in inter-patient variability. Fifty-four adult patients with advanced cancer were recruited in a prospective, multi-centre, open label dose individualization study. Patient characteristics were not shown to influence methadone response, and no significant associations were observed for methadone dose or pain score. The findings of our review of association studies for *OPRM1* A118G in advanced cancer pain demonstrate the importance of taking ancestry into account. While our sample size was small, our results were consistent with European populations, but in contrast to studies in Chinese patients, where carriers of the A118G polymorphism were associated with higher opioid dose requirements. Pharmacogenetic studies in palliative care are challenging, continued contribution will support future genotype-based drug dosing guidelines.

## Introduction

Cancers are among the leading causes of morbidity and mortality worldwide^1^ and 30–40% of cancer patients experience pain, with this rate increasing to 70–90% in patients with advanced and progressive disease^2^. As the most common and feared symptom, effective management of pain is crucial in palliative care^1^. While opioids are commonly prescribed in the treatment of moderate to severe cancer pain, inter-patient variability in response to opioids is well known^3^, and as a result many patients still experience pain or serious side effects. Recently there has been increasing appreciation that a patient’s individual genetic makeup can affect their clinical response to opioids^4,5^. Of the pharmacogenes suggested to influence the pharmacodynamics of opioids, the μ-opioid receptor gene (*OPRM1*) has shown the most evidence for clinically relevant pharmacogenetic effects on the analgesic treatment with opioids^6^.

**Figure 1:**
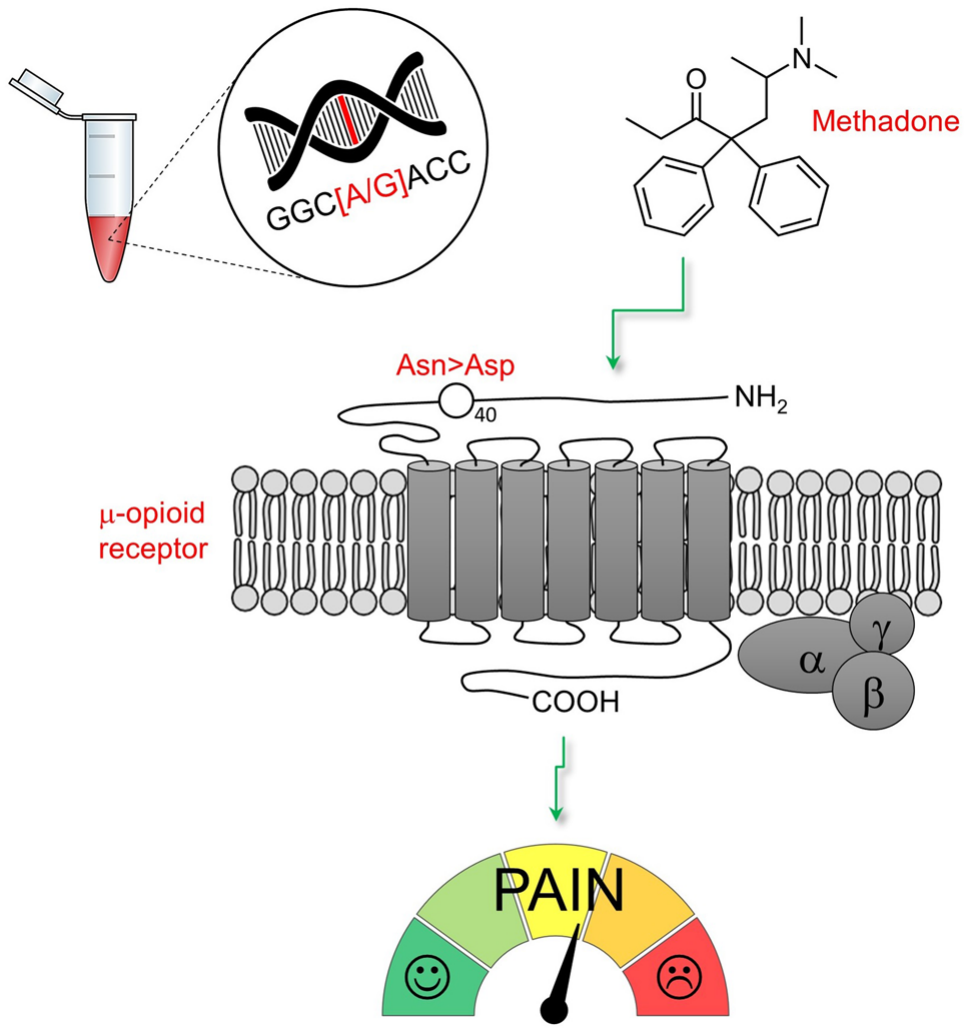
A118G polymorphism in exon 1 (rs1799971) in *OPRM1* and its consequences for opioid therapy in pain management. In this variant, substitution of a guanosine (G) for adenosine (A) results in an asparagine to aspartate amino acid change (Asn40Asp). In Chinese patients, carriers of the A118G polymorphism have been associated with higher opioid dose requirements.

The μ-opioid receptor is the primary binding site for endogenous opioid peptides and opioid analgesics, and *OPRM1* codes for this receptor. The A118G polymorphism in exon 1 (rs1799971) is the most widely studied single nucleotide polymorphism (SNP) in *OPRM1* with respect to its consequences for opioid therapy. In this variant, substitution of a guanosine (G) for adenosine (A) results in an asparagine to aspartate amino acid change (Asn40Asp), which is thought to result in the loss of a putative N-linked glycosylation site in the extracellular N-terminal domain of the μ-opioid receptor^5,7–9^ (Fig. 1).

In palliative care settings for patients with advanced cancer, individuals carrying the *OPRM1* AA genotype (wild type) have been associated with a more favourable phenotype, showing better response to opioids with lower dose, and fewer side effects^10–15^. However, some studies have shown no associations^16–23^. The opioids investigated in these studies included morphine, fentanyl, oxycodone, tramadol, tapentadol, hydromorphone, and buprenorphine. Of these studies, only one included methadone as the primary intervention for 25 patients.

Methadone is a long acting synthetic opioid that has complex pharmacokinetics^2^ and is typically only prescribed by specialist palliative care (SPC) doctors as a second line opioid, in an attempt to improve the balance between analgesia and adverse effects, and in difficult pain control scenarios^24,25^. In addition to its activity as a potent opioid receptor agonist, it also has antagonist activity at the *N*-methyl-d-aspartate (NMDA) receptor, resulting in interest for its use in clinical conditions including neuropathic pain syndromes and hyperalgesic states^24^. Methadone has many advantages including low cost, high oral bioavailability, rapid onset of effect, and a lack of active metabolites^2^, and it has been suggested that methadone should be used as a first-line opioid in the management of cancer pain^24^.

On the basis of these findings, where some studies have shown the *OPRM1* A118G polymorphism to have an effect on response to opioids, we investigated whether the A118G polymorphism of *OPRM1* may account for inter-individual variability in methadone treatment response for pain management in palliative care.

## Results

Fifty-four adult patients with advanced cancer were recruited in this prospective, multi-centre, open label dose individualization study. Complete pain scores and genotyping data were available for 46 participants (Table 1). One participant was excluded due to insufficient pain score data and seven participants did not have a sufficient blood or saliva sample size for genotyping analysis. The median (IQR) age, weight, and BMI was 62 (23) years, 68.0 (17.1) kg and 24.4 (6.0) kg/m^2^ respectively, and 27 (58.7%) patients were female. The most common cancer types included 8 (17.4%) breast, 8 (17.4%) colorectal, 6 (13.0%) cervical, 5 (10.9%) lung, and 4 (8.7%) mesothelioma cancers. Methadone was administered via the oral route twice daily with dosing titrated according to patient need by the palliative care specialist. The prescribed dose of methadone ranged from 2.5 to 50 mg twice daily. The median (IQR) number of samples obtained from each participant was 2 (3). The methadone dose was titrated as required to control pain. For participants providing multiple samples, methadone dose and pain scores were averaged across all samples.

**Table 1:** Patient characteristic variables for study population.

Patient characteristic (n = 46)		Value
Gender *n* (%)	Female	27 (58.7)
	Male	19 (41.3)
Age (years)	Median (IQR)	62 (23)
Weight (kg)		68.0 (17.1)
BMI (kg/m^2^)		24.4 (6.0)
Cancer type *n* (%)	Breast	8 (17.4)
	Colorectal	8 (17.4)
	Cervical	6 (13.0)
	Lung	5 (10.9)
	Mesothelioma	4 (8.7)
	Endometrial	3 (6.5)
	Pancreatic	3 (6.5)
	Prostate	2 (4.3)
	Multiple myeloma	2 (4.3)
	Gallbladder, bladder, liver, periauricular squamous cell carcinoma, skin cancer	1 (2.2)
Methadone daily dose (mg)	Median (IQR)	11.3 (13.9)
Pain score (Scale 0–10)		3.9 (3.2)
Genotype frequency	n (%)	A/A: 34 (73.9) A/G: 11 (23.9) G/G: 1 (2.2)
Allele frequency		A: 0.859 G: 0.141

None of the patient characteristics including gender, age, height, weight, BMI, renal and liver function were found to significantly determine the outcomes of methadone dose or pain score (Supplementary Table 1).

Genotype frequencies for the study population were AA (n = 34), AG (n = 11) and GG (n = 1) and was found to be in Hardy–Weinberg equilibrium (*p* > 0.05). The 118G allele is the minor allele across most populations, with frequencies ranging from 4% in African American samples to approximately 16% in samples of European ancestry and 40–60% in Asian samples^26^. The allele frequencies for our Australian cohort (A = 0.859; G = 0.141) were similar to those reported for the European populations of 1000Genomes (A = 0.838; G = 0.162) and ALFA (A = 0.867; G = 0.133)^27^.

Methadone daily dose and pain score are shown for each genotype in Table 2. The median (IQR) methadone daily dose was 11.3 (13.9) mg and the patient reported pain score out of ten was 3.9 (3.2). Due to the small sample size, with only one patient carrying the GG genotype, data for patients who were homozygous or heterozygous for the 118G minor allele were combined. No significant associations were observed for methadone dose or pain score, when treated as continuous variables, and no significant association was shown between genotypes and low (≤ 3/10) or high (> 3/10) pain scores (*p* > 0.05) (Table 2).

**Table 2:** Methadone dose and pain score for each genotype.

Outcome	Genotype frequency				Test statistic	*p* value
	A/A n = 34 (73.9%)	A/G n = 11 (23.9%)	G/G n = 1 (2.2%)	A/G+G/G n = 12 (26.1%)		
Methadone dose (mg/day) *Median (IQR) (min, max)*	15 (12.8) (5, 58.3)	7.7 (17.9) (3.8, 93.3)	5.0	7.6 ± 14.4 (3.8, 93.3)	*H* = 2.121	0.145^a^
Pain score (0–10) *Median (IQR) (min, max)*	3.8 (3.3) (0, 8)	4.0 (2.0) (0, 5.7)	2.3	3.9 ± 1.9 (0, 5.7)	*H* = 0.347	0.556^a^
Low pain score (≤ 3/10) *n (%)*	15 (44.1)	4 (36.4)	1 (100)	5 (41.7)	χ^2^ = 0.022	0.883^b^
High pain score (> 3/10) *n (%)*	19 (55.9)	7 (63.6)	0 (0)	7 (58.3)		

^a^Kruskal-Wallis H test.

^b^χ^2^ test for association of methadone dose or pain score with genotype A/A compared to A/G+G/G.

Table 3 summarizes the characteristics of studies from the literature on the association of *OPRM1* A11G and response to opioids for pain management in advanced cancer. χ^2^ analysis was used to determine any significant differences in allele frequencies between our study cohort and these reported studies. The allele frequencies for our cohort were similar to those reported for the studies in European populations, but significantly different (*p* < 0.001) for Chinese^13–15^ and Japanese^19,20^ patients. This ethnic variance in *OPRM1* A118G is also evident when considering the East Asian populations for 1000Genomes (A = 0.607; G = 0.393) and ALFA (A = 0.583; G = 0.417)^27^.

**Table 3:** Characteristics of studies on the association of *OPRM1* A11G and response to opioids for pain management in advanced cancer.

Study references	Study site, design and included participants	Intervention	Genotype distribution	Allele frequency	Study findings
Campa et al. (2008)^12^	137 cancer patients receiving morphine, Italy	Morphine (oral)	AA (n = 106) AG (n = 22) GG (n = 10)	A = 0.848 G = 0.152	AA genotype associated with a significant decrease in pain score from baseline compared to AG and GG
Chatti et al. (2016)^16^	129 patients with cancer pain, Tunisia	Morphine (oral)	AA (n = 98) AG (n = 31) GG (n = 0)	A = 0.880 G = 0.120	No significant association between genotypes and the dose of morphine needed for pain relief
Droney et al. (2013)^17^	249 patients with cancer pain, UK (Caucasian)	Morphine (oral)	AA (n = 183) AG (n = 58) GG (n = 8)	A = 0.851 G = 0.149	No association between genotypes and residual pain (pain score) or central side-effects in a principal components analysis study
Gong et al. (2013)^13^	112 patients with moderate to severe cancer pain, China	Opioids (morphine, tramadol, oxycodone, fentanyl)	AA (n = 44) AG (n = 50) GG (n = 18)	A = 0.616* G = 0.384	AG and GG genotype associated with a significantly higher dose than AA, and GG requiring a significantly higher dose than both AG and AA
Hajj et al. (2017)^10^	89 palliative care patients with advanced cancer, Lebanon	Morphine (IV)	AA (n = 69) AG (n = 20) GG (n = 0)	A = 0.888 G = 0.112	AG genotype associated with significantly higher dose of morphine than AA
Klepstad et al. (2004)^11^ Reyes-Gibby et al. (2007)	99 advanced cancer patients with adequate analgesia (BPI average pain score < 4), Norway Same population as above	Morphine (oral)	AA (n = 78) AG (n = 17) GG (n = 4)	A = 0.874 G = 0.126	AG genotype associated with a significantly higher pain score, GG associated with significantly higher dose Patients jointly carrying AA and Met/Met for COMT rs4680 (Val158Met) required the lowest dose to achieve pain relief
Klepstad et al. (2011)^18^	1745 cancer patients with moderate to severe pain at 17 centres including Denmark, Finland, Germany, Great Britain, Iceland, Italy, Lithuania, Norway, Sweden, Switzerland	Opioids (morphine, oxycodone, fentanyl, other opioids)	AA (n = 1335) AG (n = 385) GG (n = 25)	A = 0.875 G = 0.125	No significant associations between genotypes and opioid dose in the development (n = 1177) or validation (n = 568) sample
Li et al. (2016)^14^	59 patients with severe cancer-induced pain, China	Oxycodone (oral sustained-release)	AA (n = 23) AG (n = 28) GG (n = 8)	A = 0.627* G = 0.373	AG and GG genotype associated with a significantly higher dose than AA, with adverse effects not associated with the polymorphism
Matic et al. (2017)^28^	238 advanced cancer patients referred to a pain consultation service due to inadequate analgesia, Netherlands	Opioids (fentanyl, oxycodone, hydromorphone, morphine, buprenorphine) with 9% requiring ketamine as an adjuvant analgesic	AA (n = 192) AG (n = 45) GG (n = 1)	A = 0.901 G = 0.099	A trending increase in change of morphine equivalent dose increase from baseline was seen for 118A>G, which converted to a significant 50% higher required dose increase for those patients who also carried the COMT rs4680 (Val158Val) genotype
Matsuoka et al. (2012)^19^	41 opioid naïve patients with malignant neoplasms, Japan	Morphine (mixed routes)	AA (n = 12) AG (n = 21) GG (n = 8)	A = 0.549* G = 0.451	No association was observed between the *OPRM1* 118A>G genotype and the plasma concentration or the required dose of morphine
Naito et al. (2011)^20^	62 cancer patients receiving oxycodone in a dose escalation study, Japan	Oxycodone (oral extended-release)	AA (n = 19) AG + GG (n = 43)	A = 0.581* G = 0.419	No significant association observed in the incidence of dose escalation between genotypes
Oosten et al. (2016)^21^	339 moderate-to-severe cancer-related pain, Netherlands	Opioids (oxycodone, morphine, fentanyl, hydromorphone)	AA (n = 269) AG + GG (n = 70)	–	No association between genotypes and opioid failure, defined as rotation to another opioid or treatment with intrathecal opioids due to insufficient pain control and/or side effects, or the use of palliative sedation because of refractory symptoms associated with opioid treatment in the dying phase
Ross et al. (2005)^22^	156 cancer patients (117 controls and 39 switchers), UK (Caucasian)	Opioids (morphine, oxycodone, other opioids)	AA (n = 114) AG (n = 37) GG (n = 5)	A = 0.849 G = 0.151	No association between genotypes of patients who responded to morphine (control) vs those switching to an alternate opioid
Takemura et al. (2023)^23^	222 in-patients receiving cancer pain treatment as part of an opioid introduction or opioid rotation strategy, Japan	Opioids (fentanyl, hydromorphone, oxycodone, methadone, tapentadol)	AA (n = 81) AG (n = 74) GG (n = 67)	A = 0.532* G = 0.468	No association for those patients receiving tapentadol (n = 28) and methadone (n = 25), but a significantly smaller reduction pain score in G-allele carriers for hydromorphone (n = 67), oxycodone (n = 26), and fentanyl (n = 76) groups
Ying et al. (2016)^15^	66 Han Chinese patients with medium and severe cancer pain, China	Opioids (oxycodone, morphine, fentanyl)	AA (n = 24) AG (n = 35) GG (n = 7)	A = 0.629* G = 0.371	AG and GG genotype associated with a significantly higher opioid dose than AA

*p < 0.001 (as determined by χ^2^ test for allele frequency compared to our study).

While the results for the association of *OPRM1* A11G and response to opioids for pain management in advanced cancer appear conflicting (Table 3), all studies conducted in China^13–15^ showed the AG and GG genotypes to be associated with a significantly higher opioid dose than AA, while those studies conducted in the UK, Netherlands and European cohorts showed no association between genotypes and opioid dose requirements^17,18,21,22,28^. Studies conducted in Lebanon^10^ and Tunisia^16^ had no carriers of the homozygous GG genotype. Two studies conducted in Japan showed no significant associations^19,20^. One study comparing the effects of *OPRM1* A118G on different opioids in 222 in-patients in Japan showed no association for those patients receiving tapentadol (n = 28) and methadone (n = 25), but a significantly smaller reduction in pain score in G-allele carriers for hydromorphone (n = 67), oxycodone (n = 26), and fentanyl (n = 76) groups^23^.

## Discussion

*OPRM1* is the most studied pharmacogene associated with the pharmacodynamics of opioids, for a variety of pain conditions including experimentally induced pain, postoperative pain, chronic non-malignant pain and cancer pain. The results from several studies on the association of the A118G polymorphism on analgesia and opioid dose requirements are conflicting. Reviews on the associations of *OPRM1* A118G and experimental pain have reported study findings ranging from increased to decreased pain threshold, to not finding significance^29,30^. For post-operative pain, meta-analyses^31,32^ have indicated that the A118G polymorphism is associated with opioid requirements and adverse effects in pain treatment, however reviews have reported mixed results^29,30,33,34^. For chronic pain, reviews have identified an association between the polymorphism and chronic low back pain, but not for other types of chronic pain^35,36^. Studies on pain in advanced cancer are also disparate. A recent review by Bugada et al*.*^8^ and a meta-analysis by Yu et al*.*^37^ have suggested that while further studies are needed, the polymorphism is associated with opioid analgesia effects on cancer pain, in particular in Asian patients. Another study by Lui et al*.*^38^ on 96 ethnic Chinese patients with adenocarcinoma of the colon, rectum, or stomach who were treated with tramadol/acetaminophen combination tablets for oxaliplatin-induced painful neuropathy showed that patients with the AA genotype (n = 30) had a better analgesic effect than those with G allele variants (AG: n = 56 and GG: n = 10), with the requirement for rescue analgesia also higher for patients with G allele variants.

Only one study on pain in cancer included methadone as the primary intervention for 25 patients^23^. Association studies on *OPRM1* A118G and methadone response have been conducted in methadone maintenance treatment (MMT) settings for the treatment of heroin dependence, however the influence of this SNP on MMT outcome remains unclear^39–41^. The results of our study of 46 patients receiving methadone for advanced cancer showed no association for methadone dose or pain score which is consistent with studies conducted in European populations^17,18,21,22,28^. The results are also consistent with a recent GWAS study of 178 advanced cancer patients receiving a variety of opioids for pain, where no association was shown between *OPRM1* A118G and pain severity, opioid dose requirement or pain response^42^.

While the results of our review on association studies of *OPRM1* A118G and response to opioids in advanced cancer pain appear to show conflicting results, they do demonstrate the importance of taking ancestry into account at the individual level, where possible. Studies in Chinese populations consistently showed patients with the AG and GG genotype to be associated with significantly higher opioid dose requirements than those with the AA genotype. The recent study by Takemura et al.^23^ comparing the effects of five opioids in cancer in-patients in Japan showed a significantly smaller reduction in pain score in G-allele carriers for patients receiving hydromorphone, oxycodone and fentanyl, while no association was shown for those receiving methadone and tapentadol. The authors concluded that tapentadol and methadone may be more suitable than hydromorphone, oxycodone, and fentanyl for G-allele carriers due to their dual mechanisms of analgesic action (i.e. noradrenaline reuptake inhibition for tapentadol and NMDA receptor antagonism for methadone) and low susceptibility to *OPRM1* A118G polymorphism^23^.

It is also important to note that genetic polymorphisms of the cytochrome P450 enzymes involved in opioid pharmacokinetics can also affect response to opioid therapy^43^. There are many challenges inherent in conducting studies on the management of cancer pain. The patients’ pain is often not stable with each individual having a different pattern of escalation in pain, depending on the status of their disease, requiring individual dose titration guided by breakthrough doses and/or other background opioids. There is also significant attrition and small sample sizes are common. While we collected data across two study sites over an extended period, our sample size was small with only one patient identified as carrying the homozygous GG genotype. Another limitation of the study is the diverse mixed lineage inherent to the Australian population with participants in our study unable to be stratified into traditional distinct homogenous groups by race/ethnicity.

It is hoped that this study will provide a useful starting point for further research in this challenging field. While research conducted in this uncontrolled ‘real-life’ setting in patients with advanced cancer may be seen as a limitation, it should not preclude further research in the area. Further studies will build on the growing evidence base that will allow for the continued development of gene-drug dosing guidelines.

As the technology in pharmacogenomic testing becomes more accessible and economical^4^, clinicians will be faced with having patients’ genotypes available even if they have not explicitly ordered a test with a specific drug in mind. The Clinical Pharmacogenetic Implementation Consortium (CPIC) is an international consortium that creates and disseminates peer-reviewed, freely available genotype-based drug-dosing guidelines for clinicians that are regularly updated and referenced in ClinGen and PharmGKB^44^. The goal of the CPIC is to translate genetic laboratory test results into actionable prescribing decisions for affected drugs. A standard system for grading based on genotype/phenotype that includes a standardized system for assigning strength to each prescribing recommendation^44^. This free, online, searchable repository will be a valuable therapeutic decision support tool for clinicians as new studies become available in the future.

Of further interest is that opioids are reported to be immunosuppressive and affect the endocrine system^45^, and the μ-opioid receptor is a major element underlying pain, the endocrine system and immune function. As such, *OPRM1* might be used in the future to customize opioid therapy, not only to improve treatment and avoid side effects, but also in predicting disease progression^8,46^.

## Conclusion

The A118G polymorphism in *OPRM1* has been associated with a number of phenotypes including drug addiction, response to treatment (pain, maintenance pharmacotherapy for opioid dependence), personality traits, and response to stress^26^. We did not find an association with methadone dose or pain scores for our study, although our sample size was small and focused on the A11G polymorphism. The results of our review on association studies of *OPRM1* A118G in advanced cancer pain, suggest the importance of taking ancestry into account at the individual level, where possible. Initial dosing considerations may include accounting for the significant associations for the 118G allele and cancer pain for Chinese patients, as compared to European populations. Further studies are needed to determine whether methadone may be more suitable for pain management than other opioids for G-allele carriers due its dual mechanisms of analgesic action and thus potential lower susceptibility to the *OPRM1* A118G polymorphism. Accurate predictions of response to opioid therapy should also include consideration of polymorphisms involved in opioid pharmacokinetics. As the technology in genetic testing becomes more accessible, consideration of pharmacogenomic factors is likely to play an important role in improving patient outcomes in palliative care. Freely available online genotype-based drug-dosing guidelines for clinicians, such as CPIC, will significantly assist in expediting the translation of research findings to the clinic. While clinical studies in palliative care are challenging, we encourage continued research in the area to provide evidence to support clinicians in achieving better treatment outcomes and quality of life for their patients.

## Materials and methods

### Study participants and procedures

This is a sub-study of a prospective, multi-centre, open label dose individualization study of methadone for pain management in palliative care. Fifty-four adult patients with advanced cancer were recruited through the oncology and palliative care services of the Mater Adults Hospital (MAH) and St Vincent’s Private Hospital (SVPH) in Brisbane between 2013 and 2016. Written informed consent for genotyping analysis was obtained from all participants and ethics approval was granted by both Human Research Ethics Committees (#HREC/13/MHS/103, #HREC/13/15). Methadone was administered via the oral route twice daily according to clinical need as assessed by the SPC doctor often in conjunction with other opioids, considering current breakthrough and/or background doses. The methadone dose was recorded, including any titrations to the dose. Patient characteristics including type of cancer, liver and renal function were recorded. Due to the vulnerable nature of the population, samples were taken when it was convenient for the participant, and at each time blood and saliva was collected for the dose individualization study^47^. Pain intensity was assessed using the Brief Pain Inventory (BPI)^48^. Participants were required to rate their pain “right now” on a numerical rating scale from 0 to 10 using the BPI, with 0 representing no pain and 10 worst pain intensity (pain as bad as you can imagine) each time blood and saliva were collected. All participants who were aged ≥ 18 years, able to read and understand the patient information sheet, provide written consent, and agree to the provision of blood and saliva samples were eligible to enrol in the study. Exclusion criteria included those patients with oral mucositis, infection, or xerostomia. A sample size of 50 participants, providing two to four samples, was determined to be the minimum number necessary to generate satisfactory estimates of the structural parameters (clearance and volume of distribution) and the variance parameters (interindividual and inter-occasion variability) for non-linear mixed effect modelling (population pharmacokinetic modelling) for the dose individualization study.

### Genotyping

Genomic DNA (gDNA) was extracted from whole blood collected into EDTA tubes using an in-house salting-out method^49^ at the Genomics Research Centre, Queensland University of Technology, Brisbane. A NanoDrop™ ND-1000 spectrophotometer (ThermoFischer Scientific Inc., Waltham, MA, USA) was used to measure DNA concentration and purity before dilution to 15–20 ng/μL and storing as stock gDNA at 4 °C. Genotyping of *OPRM1* (rs1799971, 118A>G N40D) was conducted via pyrosequencing with primers designed using Pyromark Assay Design software (QIAGEN): 5ʹ CACTGATGCCTTGGCGTAC, 5ʹ GGGCACAGGCTGTCTCTC (biotinylated) and sequencing primer 5ʹ CAACTTGTCCCACTTAGAT. Pyrosequencing was performed on a QSeq platform (BioMolecular Systems) using Pyromark Gold Q24 reagents (QIAGEN). Sequencing traces were analyzed with QSeq software, version 2.1.3 (BioMolecular Systems). All genotyping was conducted by investigators blinded to sample identity. Genotypes were assigned using all of the data from the study simultaneously and not in batches.

### Statistical analysis

Deviation of Hardy–Weinberg equilibrium was tested using the chi-square (χ^2^) test (*p* < 0.05) and the observed minor allele frequency (MAF) compared with the MAF from relevant populations in dbSNP (National Center for Biotechnology Information)^27^. Clinical data are described as mean ± standard deviation (SD) or medians and interquartile ranges, as appropriate for continuous measures. Nominal variables are described as frequencies and percentages. The adequacy of each statistical test was assessed by examining residuals for heterogeneity and normality. Regression analysis was used to examine whether the outcomes of methadone dose and pain score were dependent on patient characteristics not related to the *OPRM1* A118G genotype, including age, gender, body mass index (BMI), liver and kidney function. Kruskal–Wallis H test was used to determine whether genotypes were associated with the methadone dose or pain score. For participants providing multiple samples, methadone dose and pain scores were averaged across all samples. χ^2^ analysis was used to determine significant associations for pain score, where low and high pain were categorized as ≤ 3/10 and > 3/10, respectively. Data was analyzed using IBM SPSS Statistics for Windows, version 26.0 (Armonk, NY: IBM Corp) and significance was considered if* p* < 0.05.

### Ethics approval and consent to participate

This study was performed in line with the principles of the Declaration of Helsinki. Approval was granted by the Human Research Ethics Committees at Mater Health Services (# HREC/13/MHS/103), St Vincent’s Health and Aged Care (#HREC/13/15) and Griffith University (#PHM/17/13/HREC). Informed consent was obtained from all individual participants included in the study with all samples deidentified prior to use.

### Supplementary Information

Supplementary Table 1.

## Data Availability

All data generated or analysed during this study are included in this published article (and its supplementary information files). All genotype data has been deidentified to ensure no connection to individual participants.

## References

[1] Wiffen PJ, Wee B, Derry S, Bell RF, Moore RA (2017). Opioids for cancer pain—An overview of Cochrane reviews. Cochrane Database Syst. Rev..

[2] Nicholson AB, Watson GR, Derry S, Wiffen PJ (2017). Methadone for cancer pain. Cochrane Database Syst. Rev..

[3] World Health Organization (2018). WHO Guidelines for the Pharmacological and Radiotherapeutic Management of Cancer Pain in Adults and Adolescents.

[4] Patel JN, Wiebe LA, Dunnenberger HM, McLeod HL (2018). Value of supportive care pharmacogenomics in oncology practice. Oncologist.

[5] Matic M, de Wildt SN, Tibboel D, van Schaik RHN (2017). Analgesia and opioids: A pharmacogenetics shortlist for implementation in clinical practice. Clin. Chem..

[6] Mura E (2013). Consequences of the 118A>G polymorphism in the OPRM1 gene: Translation from bench to bedside?. J. Pain Res..

[7] Kumar S, Kundra P, Ramsamy K, Surendiran A (2019). Pharmacogenetics of opioids: A narrative review. Anaesthesia.

[8] Bugada D, Lorini LF, Fumagalli R, Allegri M (1951). Genetics and opioids: Towards more appropriate prescription in cancer pain. Cancers (Basel).

[9] Wang XS (2015). Association of single nucleotide polymorphisms of ABCB1, OPRM1 and COMT with pain perception in cancer patients. J. Huazhong Univ. Sci. Technol. Med. Sci..

[10] Hajj A (2017). OPRM1 c.118A>G polymorphism and duration of morphine treatment associated with morphine doses and quality-of-life in palliative cancer pain settings. Int. J. Mol. Sci..

[11] Klepstad P (2004). The 118 A>G polymorphism in the human µ-opioid receptor gene may increase morphine requirements in patients with pain caused by malignant disease. Acta Anaesthesiologica Scandinavica.

[12] Campa D, Gioia A, Tomei A, Poli P, Barale R (2008). Association of ABCB1/MDR1 and OPRM1 gene polymorphisms with morphine pain relief. Clin. Pharmacol. Ther..

[13] Gong XD (2013). Gene polymorphisms of OPRM1 A118G and ABCB1 C3435T may influence opioid requirements in Chinese patients with cancer pain. Asian Pac. J. Cancer Prev..

[14] Li Q, Liu Y, Yu Y, Wang Y (2016). Association of analgesic effects of oxycodone hydrochloride with the A118G of OPRM1 genes in patients with cancer pain. Her. Med..

[15] Ying L, Feixue W, Sun L, Xiang L, Wang Y (2016). Effects of OPRM1 A118G gene polymorphism on the dosage of opioids in Chinese Han population patients with cancer pain. China Pharm..

[16] Chatti I (2016). Association of the OPRM1 and COMT genes' polymorphisms with the efficacy of morphine in Tunisian cancer patients: Impact of the high genetic heterogeneity in Tunisia?. Therapie.

[17] Droney JM (2013). Analgesia and central side-effects: Two separate dimensions of morphine response. Br. J. Clin. Pharmacol..

[18] Klepstad P (2011). Influence from genetic variability on opioid use for cancer pain: A European genetic association study of 2294 cancer pain patients. Pain.

[19] Matsuoka H (2012). Expression changes in arrestin beta 1 and genetic variation in catechol-O-methyltransferase are biomarkers for the response to morphine treatment in cancer patients. Oncol. Rep..

[20] Naito T (2011). CYP3A5*3 affects plasma disposition of noroxycodone and dose escalation in cancer patients receiving oxycodone. J. Clin. Pharmacol..

[21] Oosten AW (2016). Opioid treatment failure in cancer patients: The role of clinical and genetic factors. Pharmacogenomics.

[22] Ross JR (2005). Clinical response to morphine in cancer patients and genetic variation in candidate genes. Pharmacogenom. J..

[23] Takemura, M. *et al.* Comparison of the effects of OPRM1 A118G polymorphism using different opioids: A prospective study. *J. Pain Symptom Manage*. **67**, 39–49. 10.1016/j.jpainsymman.2023.09.017 (2023). 10.1016/j.jpainsymman.2023.09.017 37757956

[24] Mercadante S, Bruera E (2018). Methadone as a first-line opioid in cancer pain management: A systematic review. J. Pain Symptom Manage.

[25] Good P (2014). Therapeutic challenges in cancer pain management: A systematic review of methadone. J. Pain Palliat. Care Pharmacother..

[26] Levran O, Kreek MJ (2020). Population-specific genetic background for the OPRM1 variant rs1799971 (118A>G): Implications for genomic medicine and functional analysis. Mol. Psychiatry.

[27] National Library of Medicine. *National Center for Biotechnology. Single Nucleotide Polymorphism Database. Reference SNP report rs1799971*. https://www.ncbi.nlm.nih.gov/snp/rs1799971 (2023).

[28] Matic M (2017). Advanced cancer pain: The search for genetic factors correlated with interindividual variability in opioid requirement. Pharmacogenomics.

[29] Gray K, Adhikary SD, Janicki P (2018). Pharmacogenomics of analgesics in anesthesia practice: A current update of literature. J. Anaesthesiol. Clin. Pharmacol..

[30] Nielsen LM (2015). Association between human pain-related genotypes and variability in opioid analgesia: An updated review. Pain Pract..

[31] Ren ZY (2015). The impact of genetic variation on sensitivity to opioid analgesics in patients with postoperative pain: A systematic review and meta-analysis. Pain Physician.

[32] Zhang X (2019). The relevance of the OPRM1 118A>G genetic variant for opioid requirement in pain treatment: A meta-analysis. Pain Physician.

[33] Aroke EN, Kittelsrud JM (2019). A primer to pharmacogenetics of postoperative pain management. AANA J..

[34] Gabriel RA, Burton BN, Urman RD, Waterman RS (2018). Genomics testing and personalized medicine in the preoperative setting. Anesthesiol. Clin..

[35] Knezevic NN, Tverdohleb T, Knezevic I, Candido KD (2018). The role of genetic polymorphisms in chronic pain patients. Int. J. Mol. Sci..

[36] Yamamoto PA, Conchon Costa AC, Lauretti GR, de Moraes NV (2019). Pharmacogenomics in chronic pain therapy: From disease to treatment and challenges for clinical practice. Pharmacogenomics.

[37] Yu Z, Wen L, Shen X, Zhang H (2019). Effects of the OPRM1 A118G Polymorphism (rs1799971) on opioid analgesia in cancer pain: A systematic review and meta-analysis. Clin. J. Pain.

[38] Liu YC, Wang WS (2012). Human mu-opioid receptor gene A118G polymorphism predicts the efficacy of tramadol/acetaminophen combination tablets (Ultracet) in oxaliplatin-induced painful neuropathy. Cancer.

[39] Fonseca F, Torrens M (2018). Pharmacogenetics of methadone response. Mol. Diagn. Ther..

[40] Taqi MM, Faisal M, Zaman H (2019). OPRM1 A118G polymorphisms and its role in opioid addiction: Implication on severity and treatment approaches. Pharmgenom. Pers. Med..

[41] Oueslati B, Moula O, Ghachem R (2018). The impact of OPRM1's genetic polymorphisms on methadone maintenance treatment in opioid addicts: A systematic review. Pharmacogenomics.

[42] Yennurajalingam S (2021). Genetic factors associated with pain severity, daily opioid dose requirement, and pain response among advanced cancer patients receiving supportive care. J. Pain Symptom Manage.

[43] Vieira CMP, Fragoso RM, Pereira D, Medeiros R (2019). Pain polymorphisms and opioids: An evidence based review. Mol. Med. Rep..

[44] Clinical Pharmacogenetics Implementation Consortium. *CPIC guidelines*. https://cpicpgx.org/guidelines/. (2023).

[45] Kosciuczuk U, Knapp P, Lotowska-Cwiklewska AM (2020). Opioid-induced immunosuppression and carcinogenesis promotion theories create the newest trend in acute and chronic pain pharmacotherapy. Clinics (Sao Paulo)..

[46] De Gregori M, Diatchenko L, Belfer I, Allegri M (2015). OPRM1 receptor as new biomarker to help the prediction of post mastectomy pain and recurrence in breast cancer. Minerva Anestesiol..

[47] George R (2017). Can saliva and plasma methadone concentrations be used for enantioselective pharmacokinetic and pharmacodynamic studies in patients with advanced cancer?. Clin. Ther..

[48] Cleeland CS, Ryan KM (1994). Pain assessment: global use of the Brief Pain Inventory. Ann. Acad. Med. Singap..

[49] Chacon-Cortes D, Haupt LM, Lea RA, Griffiths LR (2012). Comparison of genomic DNA extraction techniques from whole blood samples: A time, cost and quality evaluation study. Mol. Biol. Rep..

