# Supplementary material for: The effects of OPRM1 118A>G on methadone response in pain management in advanced cancer at end of life
Source: Sci Rep. 2024 Feb 10;14:3411. doi: 10.1038/s41598-024-54009-9 (PMC10858860; doi:10.1038/s41598-024-54009-9)
Supplement: Supplementary file 1 — Supplementary Table 1. [file 41598_2024_54009_MOESM1_ESM.docx]

**Supplementary Table 1.** Multiple regression analysis for patient characteristic variables for study population for methadone dose and pain score

| Variable | Methadone dose | | Pain score | |
| --- | --- | --- | --- | --- |
|  | **B (95% CI)** | ***p*** | **B (95% CI)** | ***p*** |
| *Patient characteristics* | | | | |
| Age | -0.363 (-0.772 to 0.046) | 0.080 | -0.036 (-0.085 to 0.013) | 0.145 |
| Height | 0.763 (-2.334 to 3.861) | 0.621 | -0.063 (-0.433 to 0.307) | 0.734 |
| Weight | -1.071 (-4.54 to 2.397) | 0.536 | 0.043 (-0.371 to 0.457) | 0.835 |
| BMI | 2.545 (-7.11 to 12.199) | 0.597 | -0.121 (-1.274 to 1.032) | 0.833 |
| Gender | -14.394 (-31.211 to 2.423) | 0.091 | -0.78 (-2.789 to 1.228) | 0.437 |
| *Liver function* | | | | |
| AST | 0.087 (-0.328 to 0.503) | 0.671 | -0.002 (-0.043 to 0.503) | 0.928 |
| ALT | 0.02 (-0.242 to 0.281) | 0.879 | 0.007 (-0.019 to 0.281) | 0.608 |
| ALP | 0.031 (-0.109 to 0.172) | 0.650 | -0.007 (-0.021 to 0.172) | 0.317 |
| GGT | -0.012 (-0.09 to 0.066) | 0.760 | 0.004 (-0.004 to 0.066) | 0.324 |
| LDH | -0.015 (-0.049 to 0.02) | 0.390 | -0.001 (-0.004 to 0.02) | 0.659 |
| ALB | 0.41 (-0.973 to 1.793) | 0.549 | -0.087 (-0.223 to 1.793) | 0.203 |
| *Kidney function* | | | | |
| CREAT | -0.152 (-0.403 to 0.099) | 0.222 | -0.009 (-0.043 to 0.025) | 0.582 |
| UREA | 1.61 (-0.319 to 3.538) | 0.097 | -0.078 (-0.337 to 0.182) | 0.541 |
| eGFR | -0.379 (-1.115 to 0.358) | 0.297 | -0.051 (-0.15 to 0.048) | 0.294 |
| CREAT CL | 0.102 (-0.077 to 0.281) | 0.249 | 0.008 (-0.016 to 0.032) | 0.503 |
